# Householders’ Mental Models of Domestic Energy Consumption: Using a Sort-And-Cluster Method to Identify Shared Concepts of Appliance Similarity

**DOI:** 10.1371/journal.pone.0158949

**Published:** 2016-07-28

**Authors:** Elizabeth Gabe-Thomas, Ian Walker, Bas Verplanken, Gavin Shaddick

**Affiliations:** 1 Plymouth University Peninsular School of Medicine and Dentistry, Plymouth University, Drake Circus, Plymouth, PL4 8AA, United Kingdom; 2 Department of Psychology, University of Bath, Claverton Down, Bath, BA2 7AY, United Kingdom; 3 Department of Mathematical Sciences, University of Bath, Claverton Down, Bath, BA2, 7AY, United Kingdom; University of Reading, UNITED KINGDOM

## Abstract

If in-home displays and other interventions are to successfully influence people’s energy consumption, they need to communicate about energy in terms that make sense to users. Here we explore householders’ perceptions of energy consumption, using a novel combination of card-sorting and clustering to reveal shared patterns in the way people think about domestic energy consumption. The data suggest that, when participants were asked to group appliances which they felt naturally ‘went together’, there are relatively few shared ideas about which appliances are conceptually related. To the extent participants agreed on which appliances belonged together, these groupings were based on activities (e.g., entertainment) and location within the home (e.g., kitchen); energy consumption was not an important factor in people’s categorisations. This suggests messages about behaviour change aimed at reducing energy consumption might better be tied to social practices than to consumption itself.

## Introduction

How best to save energy is a prominent theme for research across a number of disciplines including architecture, economics, sociology, marketing, computer–human interaction and psychology. This research is pressing because most energy generation produces carbon emissions, with associated environmental impact (e.g.,[1]). While high-level changes to the energy infrastructure and the decarbonisation of energy supply might be necessary to significantly reduce negative environmental impact [2], there is also the potential for substantial carbon savings more quickly, and at lower cost, through behaviour change at the individual level. The domestic energy sector accounted for a third of the UK’s total energy demand in 2013 [3]. Whilst structural changes to a home, such as double glazing and insulation, can greatly increase building efficiency, the pooled effect of everyday householder behaviour can also have a significant impact on energy demand. As energy-consuming behaviours are repeated frequently, long term curtailment of these behaviours across the population is likely to lead to significant energy savings.

Technological approaches to domestic energy demand reduction so far have usually focused on providing energy feedback, assuming (usually implicitly) that the key barrier to behaviour change is the householders’ lack of real-time information about their consumption [4,5]. Under this information-deficit approach, technologies such as smart meters with in-home displays have been implemented in order to make consumers more aware of what they use [6,7]. While there is evidence that many householders are indeed not aware of their overall energy consumption [8], and do not have a detailed understanding of how much energy their appliances and behaviours consume [9], the evidence on whether information provision is sufficient to change consumption is mixed. Initial studies of feedback indicated energy savings ranging between 5% and 20% [6,7]. However, such figures need to be interpreted with caution. Not only have other studies found no evidence of energy savings as result of feedback [10,11], but recently Buchanan, Russo, and Anderson [12] have argued that claims of energy reduction in earlier work are potentially exaggerated and short lived. A recent meta-analysis [13] revealed that the magnitude of energy conservation reported varies with the robustness of the experimental design (with weaker studies reporting higher energy savings). Overall, then, it is far from clear that presenting information alone is sufficient to promote significant, long-lasting reductions [14–18].

The question, then, is why does energy feedback not work as intended? Current feedback mechanisms generally provide householders with their overall energy consumption in units such as kilowatt hours and/or financial cost (often estimated). While this might improve householders’ general awareness of the total amount of energy they consume [6,11], this approach makes the untested assumption that householders understand the units in which the feedback is presented, and does not take into account householders’ perceptions of where and how energy is used in their homes. Indeed, in-home displays alone offer the user little practical advice as to what behaviours they need to change to save energy. Therefore their utility may be limited as a standalone intervention.

Even if feedback systems did provide advice, this is likely to have a bigger influence on behaviour if it incorporates knowledge of the public’s understanding of domestic energy use to ensure the message is comprehensible and actionable by its recipient. This need to consider recipients’ pre-existing perceptions has been widely demonstrated across other fields such as risk communication, strategic management and usability [19–21]. An assumption underlying the present work is that interventions aimed at reducing energy use are more likely to be successful if individuals receive more detailed feedback about their behaviour in the home, in particular where and when energy is consumed. In order to do this, we need to know more about how people perceive their environment in terms of their energy consumption and how they perceive the use of energy consuming appliances.

### Previous explorations of energy perceptions

Although there has been little previous exploration of the public’s perceptions of domestic energy use, a range of approaches, both qualitative and quantitative, have been implemented to this end. One approach has focused on energy awareness–that is, knowledge of the energy consumption of different household items in relative or absolute terms. These tend to be quantitative studies, using magnitude estimation or rank ordering. Overall, these studies suggest that people are relatively poor at estimating the consumption of a single appliance but are relatively good at ranking appliances relative to one another [22–24]. However, these studies still do not tell us whether energy consumption is important to people’s perceptions of their domestic environments–merely that people can think about consumption when prompted. A good indication of this comes from Baird and Brier [23], who used two variations of a sorting task with the same participants. People were given a set of cards displaying sources of energy consumption, ranging from small domestic items to large infrastructures such as the subway. First, participants were asked to sort the items in any way the wished, and then were asked to rank-order them in terms of energy requirements. Cluster analysis was applied to both data sets, revealing large differences in the two sorting methodologies. While the rank-ordering task produced clusters of items based on similar energy requirements, when given free rein, participants clearly did not order the items in this way but rather tended to group items by similarity of function and size. This suggests that energy demand is not a major facet of how people spontaneously think about items and infrastructures that use energy.

So the question that arises is this: if people’s mental models–their internal representations–of energy are not based around energy consumption, what *are* they based around? Mental representations of external phenomena (or concepts) are thought to be formed by the process of categorising perceptual information [25]. Categorisation is a fundamental aspect of cognition which underpins the way we encode information about the world around us (e.g., [26]). A method that has been traditionally used to reveal internal representations is sorting [27]. Sorting refers to a range of techniques where participants are required to categorise items or concepts by their relative perceived similarity [28]. Sorting tasks can reveal the structures of mental representations by providing an indication of the extent to which people perceive similarities and differences between concepts [29]. As such, sorting should be an ideal method for exploring the cognitive structures mapping appliances to energy consumption [30]. Sorting tasks provide a number of methodological advantages: they are easily undertaken by those who lack the verbal skills to articulate abstract concepts [30], and they allow the natural structure of conceptual systems to emerge. Because no theoretical structure is imposed, free sorting maintains an exploratory approach, while simultaneously allowing systematic analysis [31].

The aim of the current study was thus to explore how people understand domestic energy use by revealing how they represent a variety of sources of energy consumptive appliances around the home. We employed a free card-sorting task in which each card represented an energy-consuming object. Previous explorations of energy perceptions focused on organising appliances by magnitude of energy consumption [22,24] but it is not clear whether people would categorise appliances in this way if they had not been prompted by the task, and, more importantly, whether their daily behaviour is ever guided by categorisations based on consumption. As mentioned, Baird and Brier’s [23] free-sorting task saw participants organise energy-consuming items by function and size. Because participants in that study were not asked to describe their groupings, it is unclear if those specific categorisations were actually intended by participants or whether function and size were merely correlates of the dimensions that really underpinned the participants’ sorting. Also, the items in Baird and Brier’s study had an extremely wide range of sizes, including such non-domestic infrastructures as submarines and the subway alongside small domestic appliances like a light bulb or carving knife. This obviously extreme range may plausibly have pushed participants towards categorising items based on size. Finally, while Baird and Brier included non-domestic uses of energy, we wished to confine our range to domestic energy use in order to be able to generalise findings to home energy interventions like smart metering.

The overall aim was to reveal shared patterns in the way that people categorise domestic energy consuming appliances through the sorting task. In doing so we aimed to gain insights that may help inform the effective communication of energy consumption information to end-users.

## Method

### Ethics statement

The research was conducted in accordance with the British Psychological Society ethical guidelines, and was approved by the University of Bath Social Science Research Ethics Committee (Reference number 12–155). All participants provided written consent.

### Participants

Fifty-seven participants were recruited from two different sources to undertake a card-sorting task. Table 1 displays the number of participants and demographic data collected on the two subsamples.

**Table 1:** Demographic information for the two subsamples.

	Mean Age	SD	N	Male (n)	Female (n)
Subsample 1	67.43	9.94	23	12	11
Subsample 2	24.18	7.87	34	13	21
Total Sample	41.63	23.10	57	25	32

Subsample 1 consisted of residents of social housing from Exeter, UK, who were recruited via their social housing provider as part of a larger interdisciplinary study (www.cs.bath.ac.uk/enliten). These participants were invited to take part via a letter from their housing resident involvement manager and an advertisement in the social housing provider’s quarterly magazine. The only inclusion criterion for these participants was that they were social housing tenants or leaseholders. They received £10 (and the opportunity to win supermarket vouchers worth £50 in a prize draw) for their participation in a focus group which began with the card-sorting task. The main aim of the focus group was to inform the design of the larger study, therefore the results are not described here. As the participants in sample one were typically above retirement age, a younger sample was also sought in order to improve the generalisability of the study. Subsample 2 was recruited from a university population which mainly consisted of undergraduate students who participated for course credit. This sample was recruited via online participation systems as well as through university mailing lists and advertisement posters. These participants were asked to undertake the card-sorting task only.

To inform the sample size in the present study, we examined literature on card-sorting methodology. Tullis & Wood [32] assessed the impact of sample size by implementing a sorting task with 168 participants and conducting cluster analyses on the full data set as well as with subsets of different sample sizes (from 2 to 70 participants). They compared the similarity matrices and tree structures produced in the analysis of the subsets with those from the full sample. Correlations between the similarity matrices demonstrated a negatively increasing function which reached an asymptote of 0.95 at 30 participants, at which point variance was also greatly reduced. Comparison of the resulting dendrograms revealed high similarity in cluster structure for samples of 20 or more participants. The authors conclude sample sizes of 20–30 participants are sufficient for a card-sorting study. This is likely to vary by sample homogeneity and given that we sought to recruit participants from two quite disparate sources, we decided to exceed this recommendation.

### Materials & Procedure

Participants were each presented with 44 cards and were told that each card displayed the name of ‘something that uses energy around the home’. The items named on the cards were generated in a pre-test procedure by a separate set of participants, recruited through social media (N = 19, Mean Age = 32.7, SD = 9.6), who were asked to name as many items as they could think of which consume energy within their homes. The 44 most common items were selected as stimuli for the main study. The items ranged from inbuilt domestic systems like central heating or the shower, to small appliances such as a kettle or hairdryer.

In the main procedure, a free-sorting task was implemented, in which participants were asked to arrange the cards into piles depending on how they felt cards naturally ‘went together’; participants were instructed that they could sort the cards in any manner they wished provided they made more than one pile and fewer than 44 piles. Participants were allowed to exclude cards that they did not want to categorise, and these were treated as a pile of their own during analysis. After each participant produced their piles, they were asked to give each pile a name to describe its contents.

The order in which the cards were laid out was randomised for each participant by shuffling the cards. Participants were required to sort the cards only once (as our interest was in the categorisation which felt most logical or intuitive to the participants) and were informed that they could change their minds at any point throughout the study.

### Analysis

A hierarchical cluster analysis was used to examine the extent to which there was consistency across participants in how the cards were categorised. Our approach was to take the 361 card-piles produced across all the participants and treat these piles as the units of analysis, on the principle that each pile produced had a psychological reality to the participant who produced it. As cluster analysis identifies groups of objects that are similar–defined as being close in the multidimensional space described by the measurements employed–our analysis found card-piles that were similar to one another across the participants in terms of the appliances they contained. This thereby revealed shared ideas across participants about how the items could be categorized. This was achieved by coding each pile as a series of 44 zeros and ones, describing whether each of the 44 appliances was included in that pile or not.

The hierarchical cluster analysis was run using R Version 3.1.0 [33] with Manhattan distances as the similarity measure. Ward’s method [34] was used to evaluate the distances between clusters as it is a efficient clustering method [35] which performs well with binary data [36]. Cluster membership was determined by identifying the demarcation point, at which there is an inconsistently large change in the similarity measure between clusters. The demarcation point was identified by plotting the resulting height of each agglomeration (the distances at which piles were merged on the basis of similarity) against the number of clusters and finding the ‘elbow’ in a similar manner to a scree plot in factor analysis [37]. The interpretation was confirmed by examining the dendrogram produced by the analysis. The resulting dendrogram was converted to a phylogenetic tree which graphically exaggerates the differences between clusters, making it easier to interpret. The clusters were then described by systematic examination of the names that participants gave to the piles within each cluster [30]. The most commonly occurring name was selected as the cluster label where appropriate.

### Methodological Demonstration

The methodology just described is a novel combination of existing sorting and clustering techniques, made possible by our system of coding piles as series of zeros and ones. As previous research suggests people might not hold consistent mental models of energy [38], meaning we are not necessarily expecting simple findings, we first present a simple demonstration to show that our sort-and-cluster method can indeed produce meaningful results when consistent categories do exist across people. To do this, a pilot study was conducted using exactly the same methodology and analysis described above, but with foodstuffs rather than energy appliances. Foods were used because these are highly familiar and frequently discussed. A priori it was reasonable to assume people would share numerous, well developed mental models for these, meaning sorting elements from this domain should produce distinct categorisations.

Forty-eight participants competed the pilot sorting task (35 female, 13 male, Mean Age = 25.29 SD = 13.87). Participants were given 35 cards and told that each named a different type of food. The items for the cards were generated by discussions between three researchers and were chosen to represent a variety of different factors, for example world cuisines, solids and liquids, items traditionally eaten at different times of day, and basic food groups. The items ranged from to individual ingredients (e.g. chicken or garlic) to different combinations of ingredients (e.g. ketchup, cheesecake or roast dinner).

After using the clustering approach described above, seven distinct clusters of card-piles emerged. These were groups of foodstuffs labelled as ‘meals’, ‘vegetables’, ‘protein’, ‘sweet’, ‘condiments’, ‘dairy’ and a final general cluster with no clear description. These results suggest that our sort-and-cluster method produces multiple clear groupings, which were consistent across participants, when used with a set of items people understand well. That is, where obvious categorisations exist and are shared amongst people, this method allows them to be revealed. Therefore, the extent to which the main clustering of appliances, below, similarly shows high numbers of consistent clusters across participants, will reveal the extent to which people share conceptions of energy-consuming appliances as they clearly did for food. In particular, our interest is in how many distinct clusters are revealed and which criteria are used to categorise energy appliances. Is it the case that people categorise appliances by energy consumption, and if not, how else might they do so?

## Results

**Fig 1:**
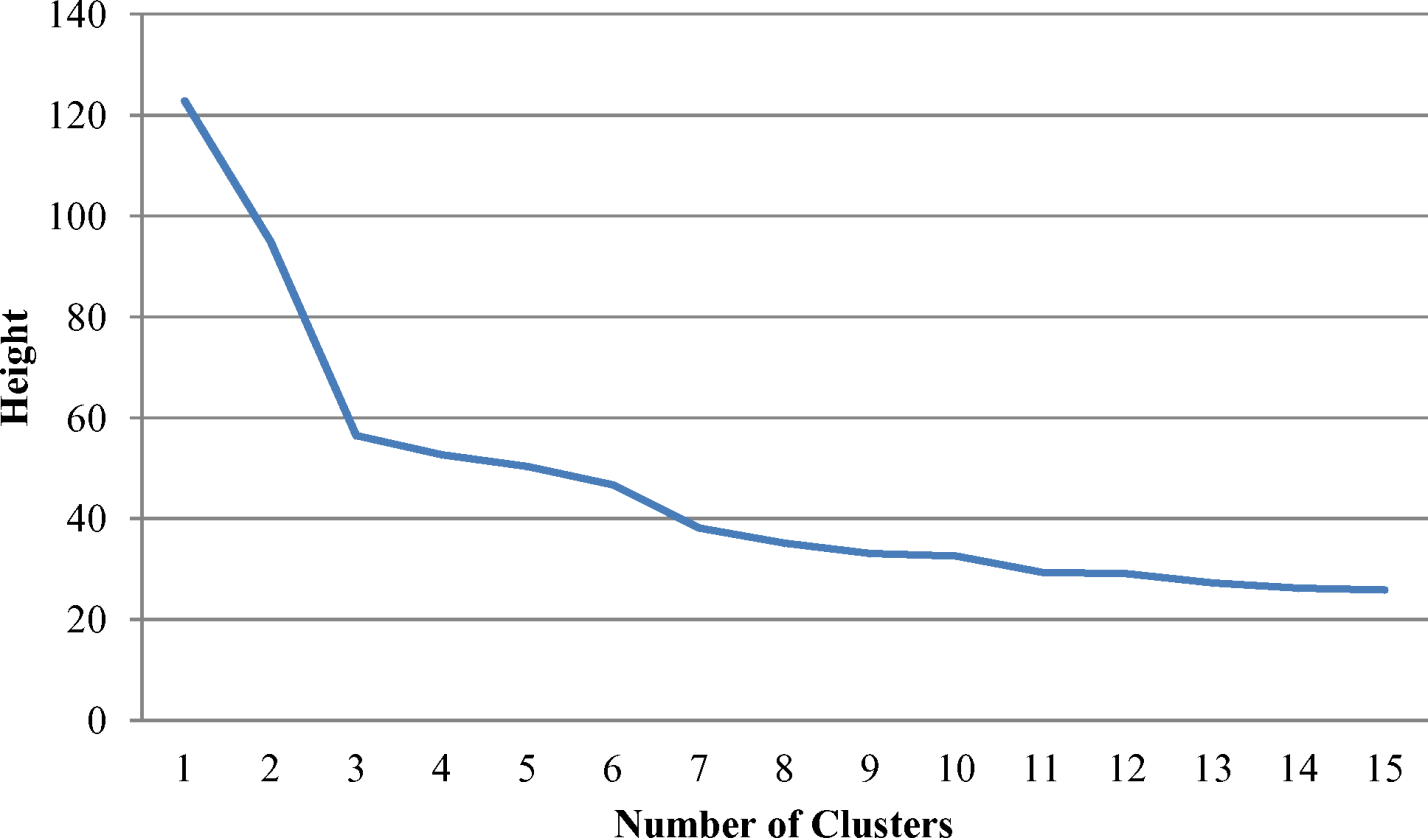
Agglomeration height plotted against the corresponding number of clusters using Ward’s method with Manhattan distances.

**Fig 2:**
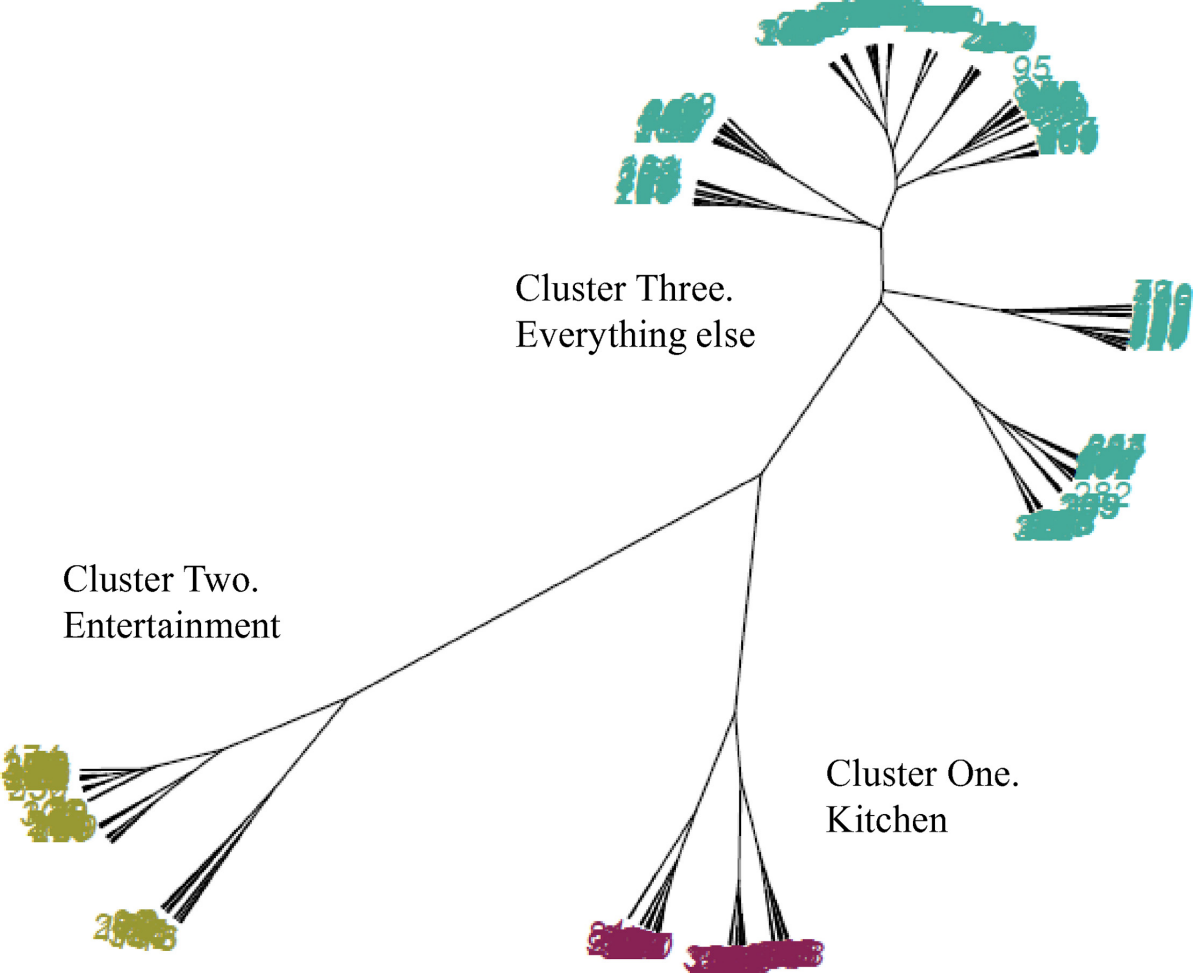
Phylogenetic tree depicting the structure of the three clusters revealed during the analysis using Ward’s method with Manhattan distances.

Fig 1 plots the height of each agglomeration against the corresponding number of cluster; the graph shows just the first 15 iterations for brevity. A very clear demarcation point (‘elbow’) can be seen indicating a three-cluster solution best fit the data–adding further clusters beyond three made little additional improvement to the clustering solution. The phylogenic tree displaying the three clusters is displayed in Fig 2.

Cluster one was labelled ‘kitchen’, as this cluster mostly contained piles of cards representing appliances used for the storage and preparation of food. Approximately 54% of the descriptions for these piles contained the word ‘kitchen’ and a further 17% mentioned ‘food’ or ‘cooking’.

Cluster two was labelled ‘entertainment’. This cluster primarily contained piles of cards primarily representing electronic devices. Fifty percent of the pile names contained the terms ‘entertainment’, ‘leisure’ or ‘living room’ by participants, other common pile names were ‘luxuries’ and ‘electronics’.

Clusters one and two were both relatively distinct, which suggests that these categorisations are consistent across participants; that is, people generally agreed that the items in each of these clusters were fairly similar to one another and different to the items in other clusters.

As is commonly the case in cluster analysis, the final cluster was larger and much less distinct than the previous two clusters. Analysis of the pile names within this cluster revealed no consistent description and so this cluster was termed ‘everything else’. Examples of the names given to piles in this cluster include ‘appearance’, ‘water users’ and ‘lighting’, illustrating the diversity of groupings that went into this cluster. The relatively large size of this cluster means that, although people mostly agreed that kitchen and entertainment appliances can be grouped together, they did not show such agreement for most other appliances.

For validation, the participants were split into various subgroups (e.g., student versus householder participants, or a set of 28 versus a set of 29 chosen at random) and the clustering repeated for each subgroup. In every case, the outcome supported the same three-cluster solution as in the above analysis. Not only do these findings demonstrate the stability of the clusters revealed in the analysis, they also show that comparable results are found with smaller sample sizes, affirming our decision to use a sample of 57 participants. Thus satisfied that three clusters was the correct solution, the final step was to move from hierarchical methods (which are best for determining the number of clusters) to *k*-means clustering, which is best for determining the content of each cluster once the number is known [35,39]**.** Given the very different clustering approaches employed by the two methods, comparison of cluster membership across the two methods should provide further reassurance of the validity of the clusters revealed. Sure enough, analysis of the piles within the three final *k*-means clusters revealed they converged completely with those revealed by Ward’s method. Furthermore 97.83% of the cases (card-piles made by participants) were classified in the same clusters by both methods.

### Analysis of the items within clusters

So far, the clusters have been described by the names that participants gave to the piles of appliance-cards they produced. But for practical purposes, we are interested in the items within those piles. Therefore we investigated the frequency with which each appliance appeared within each of the three clusters ‘kitchen’, ‘entertainment’ and ‘everything else’.

**Fig 3:**
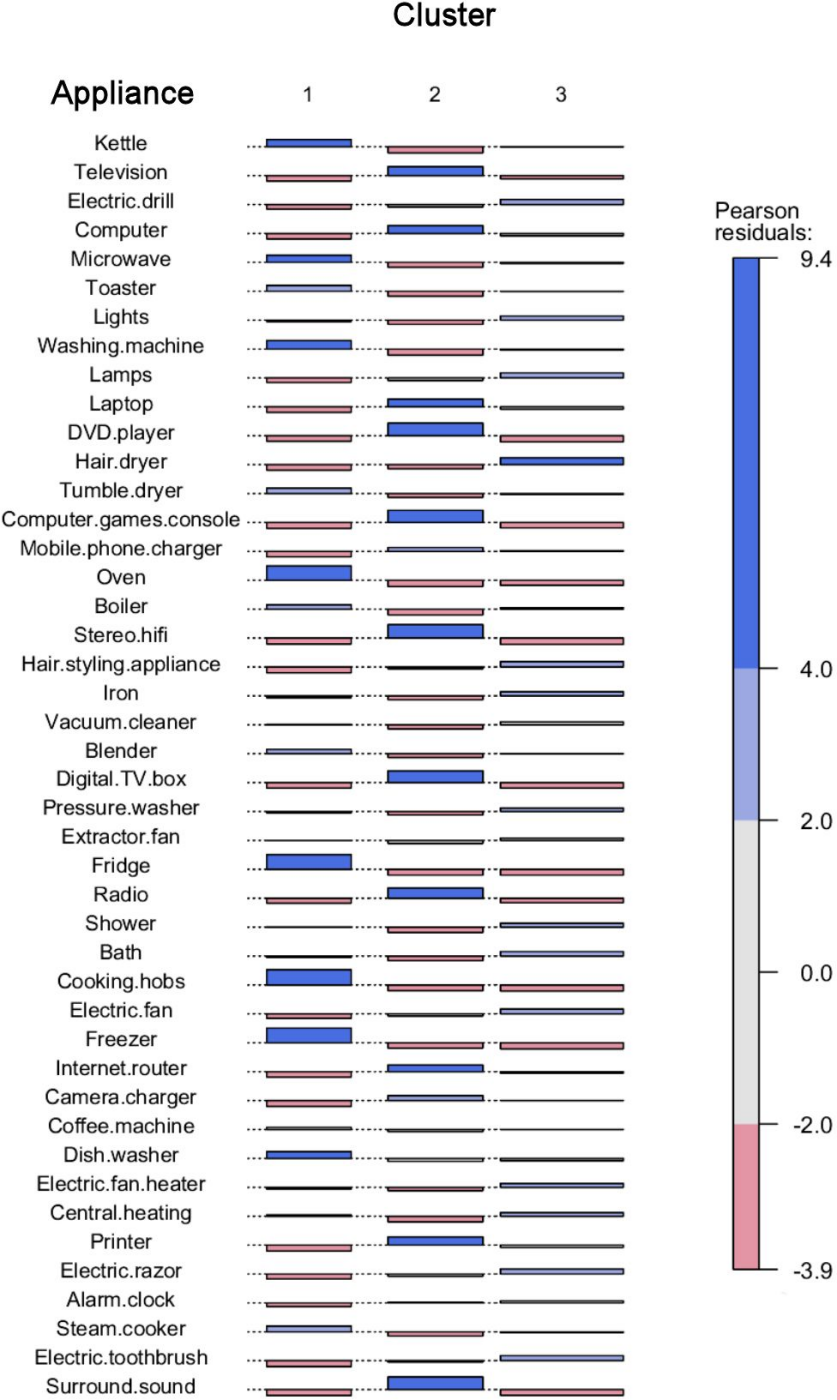
Cohen-Friendly association plot displaying the extent to which appliances became associated with the three clusters.

Fig 3 displays a Cohen-Friendly association plot which shows the proportional distance between observed and expected frequencies of occurrence within each cluster for each appliance. The rationale for this analysis is the null hypothesis that, if there were no structure to people’s sorting, the piles of cards they produced would put appliances randomly into the three clusters. The extent to which an appliance appears within a cluster more often than chance would suggest is shown in this plot by a box that stands above the baseline and is shaded blue; an appliance that appears in a cluster less often than chance would suggest is shown by a box below the baseline and is shaded red.

As Fig 3 shows, the items that were significantly associated (χ²>2.0, p < .001) with Cluster one (kitchen) were, in order of χ² magnitude: cooking hobs, fridge, freezer, oven, washing machine, kettle, dishwasher, microwave, toaster, steam cooker, tumble dryer, blender and boiler. All appliances are typically found within a kitchen. The inclusion of large appliances like washing machine, tumble dryer, dishwasher and boiler suggest that participants create the piles within this cluster based on the physical location of the kitchen rather than solely the practice of food storage and preparation. It is also notable that these large appliances were mixed with small appliances such as kettles, suggesting location and/or practice are more important than size (cf. [23]).

The items that were significantly associated (χ²>2.0, p < .001) with Cluster two (entertainment) were, in order of χ² magnitude: stereo system/HiFi, DVD player, surround sound, computer games console, digital TV box, radio, television, printer, computer, laptop, internet router, camera charger and mobile phone charger. The items are all electronic devices used to facilitate entertainment and leisure (although many could also facilitate work).

The items significantly associated with Cluster three (everything else) were as follows: hair dryer, hair styling appliance, electric drill, lamps, electric razor, electric toothbrush, electric fan, bath, lights, iron, shower, electric fan heater, central heating and pressure washer. On the whole these items do not fit in to any obvious single category, even though statistically they fit together, confirming the lack of definition in this cluster.

As can be seen in Fig 3, the Pearson residuals statistics for the items in the kitchen and entertainment cluster are considerably higher (χ²>4.0 for 8 items and 11 items respectively) than for the general household cluster (χ²>4.0 for one item only). This further supports the interpretation that the former two concepts are more consistent and clearly defined than the latter.

### Comparison of results across gender

In order to compare results for men and women, the data were split by gender and the initial cluster analysis was repeated for each dataset separately. Inspection of the resultant dendrograms and agglomeration schedules revealed a three cluster solution fit the data in each case. In order to confirm that the three clusters were similar in content to those produced in the main analysis, the names given to the piles within the clusters were examined. For men, 83% of the pile descriptions in Cluster one contained the words ‘kitchen’, ‘food’ or ‘cooking’ and 73% of the pile descriptions in Cluster two included the words ‘entertainment’, ‘leisure’, ‘living room’ or ‘luxuries’. For women, 58% of the pile description included the words ‘kitchen’, ‘food’ or ‘cooking’ and 71% of the pile descriptions in Cluster two included the words ‘entertainment’, ‘leisure’, ‘lounge’ or ‘luxuries’. The similarity of the clusters produced across genders further validates the stability of the clusters revealed in the main analysis. It is interesting to note that the number of piles in each cluster differed for men and women, While the number of card piles in each cluster was similar for men and women (N’s = 15 and 17 respectively), a larger difference was found for the ‘kitchen’ cluster. This cluster contained 12 card-piles for men, and 26 card-piles for women, hinting at a greater tendency for women to categorise energy consumption sources in this way.

## Discussion

The aim of this study was to explore the public’s perception of domestic energy use through their categorisation of energy-consuming appliances. Specifically, we wanted to know how many consistent categorisations would be revealed, and what criteria participants used to make these. The clustering, validation and association analyses presented here provide support for the existence of three stable and robust clusters of appliance groupings. The ‘kitchen’ and ‘entertainment’ clusters were relatively distinct, suggesting that these represent commonly occurring categorisations that are agreed upon across participants. The individual appliances most commonly associated with these clusters clearly corresponded with the category description. The ‘everything else’ cluster was relatively large and the items most often found within these piles showed no clear patterns. This suggests that there was substantial disagreement amongst people about how most appliances can be categorised. What is notable about the results is that so few distinct clusters were revealed. If most participants in our main analysis had agreed that, say, baths and showers belonged together, whilst also not belonging with all the other appliances, the method would have revealed this as a distinct cluster. The fact that a greater number of distinct clusters were not revealed during the analysis suggests that the participants do not hold consistent mental representations of which household appliances sit together. These findings contrast with those of the pilot study, in which many distinct categorisations of food were revealed. This consistent categorisation of foodstuffs suggests people share consistent, well developed mental representations of food as a concept. While the sorting methodology thus has the potential to reveal detailed categorisations if they exist, this apparently was not the case for the energy appliances data, thus suggesting that people only have a rudimentary shared mental model of how energy-consuming appliances relate. Of particular interest is the finding that consumption was not a consistent factor in people’s groupings.

The interpretation that people may have ill-formed taxonomies of appliances fits nicely with earlier research demonstrating that householders do not understand domestic energy consumption very well [6,22,40]. Furthermore our findings are in line with the conclusion that people do not hold consistent mental models of energy as a concept. Chisik [38] similarly found little evidence of cohesion in his participant’s mental model of electricity. This is perhaps related to the frequently noted point that energy is ‘doubly invisible’ [41]; we do not witness it entering our homes, and its consumption is tied up ‘inconspicuously’ in our everyday routines [42]. Indeed, evidence suggests that energy is consumed with little awareness of the quantity or is impact on the environment (e.g. [43]). In contrast, the consumption of food, which we also used here, is arguably far less ‘invisible’. Whilst energy consumption is hidden within other activities, and is a means of achieving a particular behaviour rather than the goal, people consume food more consciously, for pleasure or to relive hunger. The acquisition of food for consumption is more often prominent than it is for energy (going to a supermarket vs. electricity entering the home via hidden wires). Therefore the invisible nature of energy may result in poorly developed collective mental models of domestic energy consumption. Speculatively, it would be interesting to see where water lies between these two points. Its entry to the home is as easy as is the entry of electricity and its use is again a means to an end rather than a goal, but it is by nature more tangible and visible.

The descriptions people gave their appliance-groupings and the items within those groupings indicate that collectively, people categorise some appliances in terms of activities (entertainment and cooking) and, perhaps to some extent, location (kitchen). These results are comparable to those of Baird and Brier [23] who concluded their participants grouped sources of energy use by function, given that function relates to the activity for which something exists. Baird and Brier also concluded that participants sorted by size, whereas there was little evidence that this is a common sorting criteria across our participants. This inconsistency could be explained by the fact that in the present study we used more objective means of interpreting the cluster criteria (i.e. systematic analysis of pile-names) and, particularly, used items that were of a similar order of magnitude in size.

The way in which participants in the present study categorised domestic appliances draws parallels with social practice theory [42]. Practice theories generally suggest that domestic energy use can be best understood as social practices, which include bundles of more specific habits and behaviours, and emerge from interactions between materials (objects), competencies (skills), and images (meanings) [42,44–46]. Under this approach, the activities that are relevant to energy consumption and the ways in which they are carried out are seen as the best way to understand energy behaviour–perhaps again echoing our earlier points about energy being a means to an end. The present study is novel in the fact that it directly suggests that people categorise energy-consuming appliances within their homes in this way. Some might say this seems intuitive, but we would argue on the contrary that it was equally possible that participants would use different criteria, and instead categorise appliances into groups of high and low energy consumption, essential and non-essential appliances, or group them by size (cf. [23]). Although some participants did organise their piles in this way, in most cases they chose not to do this.

Given the way some of our groups hint at social practices, one might expect other types of energy-relevant practices to be revealed by the analysis, for example laundering or comfort [42]. Indeed one might expect most people to agree that washing machines and tumble dryers belonged together and not with other appliances. The fact that other practice-relevant groupings were not revealed may suggest that there is something more salient about entertainment and cooking than other practices, for example they are arguably both associated with pleasure. However this conclusion is somewhat speculative for now.

Taken together, our interpretations have implications for policy and for how information about domestic energy use is communicated. Behaviour-change policy categorisation can often be at odds with how people perceive and understand behaviour [47]. For example, sustainability policy targets ‘water-using behaviours’ as a construct, but work by Pullinger, Browne, Anderson, and Medd [48] found this categorisation to be fairly meaningless to householders. Our findings suggest that focusing on the activities of entertainment and cooking themselves–rather than the more vague concept of ‘energy use’ or the more specific concept of an individual appliance–may be more meaningful to householders. The categorisations revealed in the present study could thereby provide a useful frame for intervention designers. Information of about domestic energy consumption and messages about energy-use reduction could be presented in terms of these shared representations. For example, presenting energy feedback to householders in a way that corresponds with their existing representations, i.e. in terms of entertainment or things in the kitchen, may help householders to better internalise the feedback and become more aware of their own energy use in a meaningful way. On the other hand, our findings suggest that other types of mental appliance categories are much less likely to be shared across people, and that therefore it might be necessary for feedback on other appliance behaviours to be based around individual high-consumption appliances, or on groups of appliances that are seen as common by each individual householder. Further exploration is needed and these assumptions should be formally tested on new samples before concrete recommendations can be made. To this end, future research should compare the energy consumption of households exposed to traditional feedback interventions (for example, those that simply present end-users with their total energy consumption), with practice-based interventions or disaggregated appliance interventions. Practice-based interventions could feed back householders’ energy consumption in terms of meaningful activities (e.g. the amount of energy consumed cooking) and suggest using less energy consumptive alternative materials (e.g. heating food in a microwave or convection oven rather than a traditional oven) or ways of carry out the same activities in a less energy consumptive manner (e.g. bulk cooking).

Finally, although it is assumed that categorisation has implications for behaviour [25], we cannot infer how the public perceive dynamic aspects of these elements within the home. For example we have not explored mental models of how the appliances themselves work or how they are used. Further work is needed to fully understand perceptions energy relevant practices and how they can be best influenced. Despite this, the present study offers a unique contribution by revealing how people perceive the energy consuming appliances in their environments through categorisation. If we are to communicate information to people about their environment and encourage behaviour change, it is vital that we do so in way that takes into account their existing perceptions of energy consumption in a meaningful way.

### Conclusions

The present study has demonstrated that there are relatively few shared ideas about which energy consumptive domestic appliances are conceptually related. Furthermore, where there was consistency, people conceptualised these elements as activities and locations within the home. These findings suggest that behaviour change may be best realised by communicating with householders in a way that is congruent with their existing understanding of the things that consume energy within their homes, for instance in terms of practices rather than overall consumption.
